# Stress distribution of multiple implant-supported prostheses: Photoelastic and strain gauge analyses of external hexagon and morse taper connections

**DOI:** 10.4317/jced.59288

**Published:** 2022-03-01

**Authors:** Ana-Beatriz-Bueno-Carlini Bittencourt, Erica-de Oliveira-Paiva Rezende, Marcio Campaner, Sandro-Basso Bitencourt, Daniela-Micheline dos Santos, Aldiéris-Alves Pesqueira, Marcelo-Coelho Goiato

**Affiliations:** 1 Postgraduate student, Department of Dental Materials and Prosthodontics, School of Dentistry, Aracatuba, São Paulo State University (UNESP), Aracatuba, Brazil; 2 Graduate student, Department of Dental Materials and Prosthodontics, School of Dentistry, Aracatuba, São Paulo State University (UNESP), Aracatuba, Brazil; 3 Professor, Department of Dentistry, University Center of Espírito Santo-UNESC, Colatina, Brazil; 4 Professor, Department of Dental Materials and Prosthodontics, School of Dentistry, Aracatuba, São Paulo State University (UNESP), Aracatuba, Brazil

## Abstract

**Background:**

To evaluate the stress distribution of three-element prostheses on two different implant systems (External Hexagon (EH) or Morse Taper (MT)) and with two different retention mechanisms (screw-retained or cemented), by photoelastic analysis and strain gauge analyses.

**Material and Methods:**

Four photoelastic and 24 strain gauge models of a partially edentulous maxilla were made and were divided in four groups according to connection and retention system: Group I (EH-C) – external hexagon+cement-retained prosthesis; Group II (EH-S) external hexagon+screw-retained prosthesis; Group III (MT-C) – morse taper+cement-retained prosthesis; Group IV (MT-S) – morse taper+screw-retained prosthesis. The implants were installed in the axial position, the first in the region of element 15 and the distal implant in the region of element 17. Loads of 100 N were applied on the occlusal surface of the prosthesis for 10 seconds. For the photoelasticity analysis, photographic images were taken and were evaluated according to the number of high-intensity fringes. For the strain gauge analysis, the strain gauges were positioned on the marginal crest of the implants and on the apical region, being numbered for analysis of the stress distribution in each region. The electrical signals were captured and processed by specific software.

**Results:**

Higher concentration of tension was observed in the apical region of the implants and mainly in the distal implant, where the formation of fringes was higher. The microstrain values obtained for each group were similar: EH-C (454±18,3 µɛ); EH-S (469±94 µɛ); MT-C (466±49,8 µɛ); MT-S (460±36,6 µɛ). It was observed that apical position had higher stress concentrations for all analyzed groups.

**Conclusions:**

The different connections and fixation mode did not interfere in the amount of tension generated in the tissue adjacent to the implant, also the region that generated the greatest amount of tension was in the apical region of the anterior implant.

** Key words:**Dental implants, biomechanics, fixed prosthodontics.

## Introduction

Rehabilitation with dental implants in maxillomandibular regions has been scientifically well documented over the years (1,2). The technique was introduced by Branemark and became a gold treatment for the rehabilitation of partially or totally edentulous individuals (3). For these cases, the long-term success is associated with a precise adaptation between the prosthetic components, esthetics, balance of the stomatognathic system, and resistance to masticatory movements (4).

Several types of connections were developed over the years, aiming to improve mechanical, aesthetic, and biological properties. The external hexagon (EH) and the Morse taper (CM) connection are the most used connections, however there is no consensus on which type of connection presents the lowest biomechanical risk for implant-supported rehabilitations (5). Studies have been reported the presence of micromovements of the EH connection, due to the size of the hexagon, low resistance to masticatory loads, larger microgaps, alveolar bone resorption, and failure of the rehabilitation procedure (6-8). The CM connection is frequently used in rehabilitations, due to its excellent sealing capacity, better stress distribution, greater stability between the prosthetic component and the implant, and due to the reduction of micro spaces at the interface (6,8,9).

When planning a rehabilitation, the fixation system of the prosthesis on the implant must be taken into account, and it can be screwed or cemented. Screwed systems are generally used in patients completely edentulous, due to repairable and facility to remove for cleaning and maintaining the health of periodontal tissues. Although, some studies have reported several complications, such as screw loosening, higher stress concentrations, less force dissipation, and screw fracture (1,5,10,11). Cemented prostheses, on the other hand, are generally indicated in single cases, in which aesthetics are essential. The cemented system presents lesser marginal misfits and higher dissipation of masticatory forces. In addition, they have simplified laboratory steps, satisfactory occlusion and they are ease of repair when compared to screw-retained prostheses (1,5,10). Due to this, it is important to understand the forces that affect implant-supported prostheses, to reduce complications and maintain integrity at the implant/bone interface. Therefore, it is essential to optimize the distribution of masticatory load within physiological limits so that tissue response is not adverse and that system failure does not occur (12-14).

Photoelasticity analysis is used for stress distribution evaluation of implant-supported prostheses. This methodology simulates the clinical condition of transmission of forces generated on the prosthesis during function and, consequently, transmitted to the implant and adjacent bone. It also allows the assessment of the points of greatest incidence of this force (4,15-18). Another methodology used to assess the biomechanical behavior of implants is strain gauge analysis. The application of this method is based on the use of electrical resistances, which can be used either *in vivo* or *in vitro* under static or dynamic loads (19). These resistances are very sensitive and assess the elastic deformation of the area where they are fixed. Some authors (20,22) combine photoelastic and strain gauge analyses to assess tensions around the prosthesis/implant/bone system.

During the planning of implant-supported prostheses, the connection and retention system may vary according to the edentulous space. In addition, few studies have compared the biomechanics of multiple prostheses using either external hexagon or Morse taper connections, being screwed or cemented, using the methodology of photoelasticity and strain gauge analyses. Thus, the aim of this study is to evaluate the stress distribution of implant-supported prostheses with different types of connection (external and internal connections, screw-retained and cement-retained prostheses) in multiple 3-element crowns under compression forces, using photoelastic and strain gauge methods. The first null hypothesis is that there will be no significant difference in the stress distribution around external hexagon and morse taper implants comparing the screw and cemented retention systems on both methods used. The second null hypothesis is that, independently of the strain gauge position, no significant difference in the stress distribution will be verified.

## Material and Methods

-Groups distribution

Four groups were developed for this study, according to connection and retention system: Group I (EH-C) – external hexagon+cement-retained prosthesis; Group II (EH-S) external hexagon+screw-retained prosthesis; Group III (MT-C) – morse taper+cement-retained prosthesis; Group IV (MT-S) – morse taper+screw-retained prosthesis. A 3-element prosthesis was used for all groups. Dental implants (Biofit, DSP) with the same dimension (4x11.5) were used for all groups. For photoelastic analysis, 1 sample of each group was produced, while for strain gauge analysis, 5 samples of each group were fabricated.

-Sample’s fabrication

A same prototype of a maxilla with missing teeth 15, 16 and 17 was used to fabricate all samples. Four samples (n=1) were fabricated of photoelastic resin (PL-2, Vishay, Micro-Measurements Group) and 24 samples (n=6) of polyurethane resin (F160 Axson Brazil). An artisanal silicone (Sapeca artesanato, Brazil) was used to obtain a cast of a type IV dental stone (Durone, Dentsply Inc.) for sample’s fabrication (23). The dental stone replica was drilled in the region of teeth 15 and 17, by using a parallelometer to standardize the insertion on its long axis. Implant analogues (EH or MT) were screwed to the corresponding transfer (DSP Biomedical), and placed on the perforations. The transfers were attached to each other by using dental floss and acrylic resin (Duralay Reliance Dental). Artisanal silicone was again used to obtain an impression of the dental stone cast with the implant analogues and transfers for the preparation of the photoelastic and polyurethane samples (23). The respective implants of each group were attached to their transfers on the silicone matrix before manipulation of photoelastic and polyurethane resin.

The photoelastic resin was handled according to the manufacturer’s instructions and inserted into the silicone cast with the implants. The entire assembly was submitted to a 40 lbf/pol2 pressure to avoid internal bubbles. The photoelastic cast was separated from the silicone after polymerization and polished with ﬁne-grit abrasive paper of different granulations #300, #400, #600 and #1200 (Buehler). For the strain gauge analysis, the same silicon previously described was used, where the respective implants were positioned and later filled with the F160 polyurethane resin (F160 Axson Brazil) (23).

The fixed prostheses were made of Ni-Cr alloy were used. Multiple united implant-supported fixed prostheses were made corresponding to the second premolar, first molar and second molar. For the screwed groups, the multiple implant-supported prostheses were screwed to the implants with a torque of 20 N, using a digital torquemeter (Lutron TQ-8800, Lutron Electronic Enterprise, Taiwan). For the cemented groups, the crowns were cemented using a zinc oxide non-eugenol cement (Temp-Bond NE, Kerr), under a load and time according to the manufacturer.

-Photoelastic analysis

A glass container with mineral oil was positioned between a polarizing filter and an analyzing filter. A light diffuser was attached to the polarizing filter, which allowed a white light source (Photoflood, GE Ligthing, General Electric Co, Nela Park, Clevelland, OH, USA) to fall evenly on the container with the photoelastic model. Between the polarizing filter and the analyzer, two ¼-wave plates were interposed. The filter analyzer was coupled to a digital camera (Rebel T5i, Canon) to capture the images and transferred to a computer for qualitative analysis by using imaging software (Adobe Photoshop CS6; Adobe Systems) for the visualization, comprehension, and interpretation of locality and intensity of the tensions distributed around the implants and bone tissue.

Photographic recordings were initially made without loading to verify the absence of stresses in the photoelastic casts. Then, loads of 100N were applied to fixed and standardized points on the occlusal surfaces of all crowns at the same time in the universal testing machine (EMIC DL-3000, São José dos Pinhais, Paraná, Brazil), which was programmed to transmit loads over 10 seconds (17).

The images obtained were classified according to the number of fringes and concentration of tension of each sample. For the number of fringes analyses, it was verified fringes of moderate (green-red) and high tension (green-pink). All images were evaluated by the same operator.

-Strain gauge analysis

Voltage measurements were performed in six distinct regions. Two strain gauges were bonded horizontally in the mesial and distal region of each implant directly on the marginal crest of the model and one in the apical region of each implant (21,23) (Table 1).

**Table 1:**
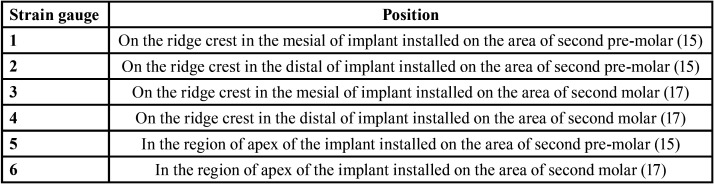
Positions of the strain gauges, according to the regions on each implant.

The strain gauges were conﬁgured into a one-quarter Wheatstone bridge and the data were transferred through a data acquisition system (ADS2000; Lynx Tecnologia Eletronica Ltd.) and processed by specific software (AqDados 7; Lynx). Each test was performed five times on each sample and the stress values were recorded in microstrains. Each test was performed only after the microstrain values were measuring zero, to verify the absence of plastic deformation.

-Statistical analysis

The data of strain gauge analysis was evaluated using one-way analysis of variance (ANOVA). Tukey test was used as a post-hoc for the analysis of the strain gauge position, with a significance of 5%.

## Results

Photoelastic analysis

**Figure 1:**
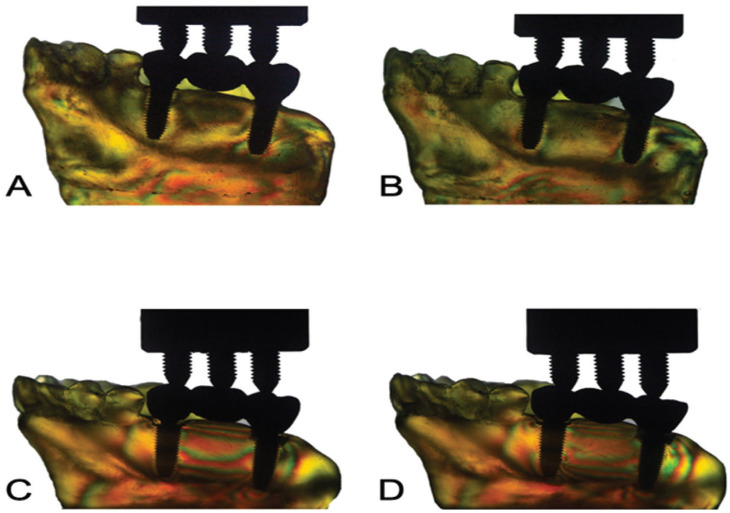
Stress distribution of each group by photoelastic analysis.Legend: A) Group I (EH-C); B) Group II (EH-S); C) Group III (MT-C); D) Group IV (MT-S).

Overall, all groups presented similar formation of fringes (Table 2). Higher concentration of tension was observed in the apical region of the implants and mainly in the distal implant, where the formation of fringes was higher (Fig. 1).

**Table 2:**
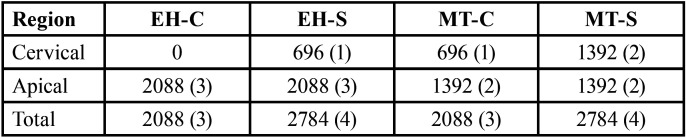
Corresponding voltage values (transition between green/pink = 696 kPa) to the number of fringes, according to each evaluation group and region.

-Strain gauge analysis

There was no statistical significance for the ANOVA test, among the groups evaluated (*P*=0.976). The microstrain values obtained for each group were similar: EH-C (454±18,3 µɛ); EH-S (469±94 µɛ); MT-C (466±49,8 µɛ); MT-S (460±36,6 µɛ). For the ANOVA of the straing gauge position, a statistical significance was verified (*P* < 0.001). Table 3 shows the microstrain values according to strain gauge position on each group.

**Table 3:**
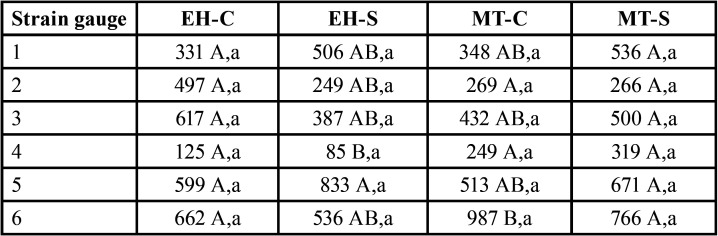
Microstrain values according to strain gauge position.

Lowercase letters represent statistical difference between cemented versus screwed rehabilitation protocols in relation to the same strain gauge position (*P* < 0.05), that is, (1x1; 2x2; 3x3; 4x4; 5x5 and 6x6). Uppercase letters represent a statistical difference (*P* < 0.05) between the different locations of the strain gauges within the same group, represented respectively by the colors blue for cemented groups and red for screwed groups.

It was observed that positions 5 and 6 (apical) had higher stress concentrations for all analyzed groups. It was observed that there was a statistical difference in position 6 within the MT-C Group. For the EH groups, the strain gauges at position 4 had the lowest stress concentration values than the other regions (Table 3).

## Discussion

The first null hypothesis was accepted, since no significant difference in the stress distribution was verified when comparing all groups. However, there was no evidence to accept the second null hypothesis, since significant difference was verified according to the strain gauge position.

The present study focused on the distribution of stress in two forms of prosthetic fixation in 3-element multiple crowns with two implant connection systems. This type of rehabilitation is well established as a treatment option for partially edentulous patients (1,2,4). Even with high success rates, complications can occur in the face of high stress where the bone begins to resorb due to its distribution capacity limit (4). In view of this, the different forms of prosthetic fixation available should be evaluated in order to minimize the tension formed.

In the present study, numerical differences were found in the generation of stresses between cemented and screwed prostheses, with cemented prostheses showing lower stresses in the evaluated regions, when subjected to axial load in the photoelastic analysis. In EH implants, the cemented prostheses tend to generate less tension regions compared to screw-retained prostheses (4). This can be attributed to the fact that the cement occupies the space between the prosthesis and the abutment, absorbing the tension generated and distributing it more evenly. When the MT implants were joined, the tension values were similar to those of the EH system. This similarity is due to the inclination that was given to these implants during their installation. This corroborates the studies by Goiato *et al*. (9,24) who found that 3-elements crowns in MT implant connection system had more high intensity fringes. According to the authors, the highest stress values after the union of screwed MT implants were due to the likely inclination that was given to these implants during installation, as the minimum lack of parallelism can influence the stress concentration caused by the structural characteristic of this system of connection. Goiato *et al*. (24) reported that when the implants were inserted into casts, with the aid of a device to promote parallelism between the implants, the CM System had a smaller amount of high intensity fringes compared to the HE System. However, it is clinically difficult to achieve perfect parallelism between implants (9).

Regarding the photoelastic analysis, the screwed and cemented prostheses showed a higher concentration of tension in the apical region of the implants. When evaluated by position, the tension distribution did not show great variation in the long axis of the implant, with only the EH-S group in the distal position of the implant 17 being a lower tension value and the highest value was recorded in the apical region of the implant MT- C. This fact is in agreement with a previous study (25), in which stress distribution in implant-supported prostheses with HE and CM connections were evaluated and a high stress concentration at the apex of the implant when subjected to axial load was found. Canay *et al*. (26) observed that the distribution of stresses around installed implants subjected to loads of 100 N in the vertical direction and 50 N in the lateral direction, appeared in the cervical and apical region, corroborating the present study. In addition, it was also evaluated in the study carried out by Borges *et al*. (27), who analyzed that the higher apical tension is not only due to bone density, but the knowledge of bone density values, both alveolar and basal. It is worth noting that the most superior areas in the maxilla, the basal bone, had greater density compared to the areas located in the alveolar bone.

Guichet *et al*. (28) carried out a comparative study between cemented and screw-retained crowns on three implants by photoelastic analysis and they concluded that cemented prostheses presented a homogeneous stress distribution in their parts when compared to screw-retained prosthesis. In this way, Çehreli *et al*. (12) evaluated the stress distribution by photoelasticity and strain gauge analyses of EH implants on two different types of prosthetic fixation (cemented and screwed), and also did not observe differences in stress generation either to axial or oblique forces.

The limitations of this study are restricted to be an *in vitro* study, due to greater control during the tests and the absence of adversities found in a clinical study. Furthermore, different shapes and sizes of implants and the use of an oblique load could influence and enrich the present study.

## Conclusions

Despite the cemented crowns on EH implants having presented lower tension for the tests; all systems are biomechanically similar and can be used for partial rehabilitations in a safe way. This choice will depend on the clinical characteristics of the patient and the criteria of the dentist.
